# New Vegetable *Brassica* Foods: A Promising Source of Bioactive Compounds

**DOI:** 10.3390/foods10122911

**Published:** 2021-11-24

**Authors:** Pilar Soengas, Pablo Velasco, Juan Carlos Fernández, María Elena Cartea

**Affiliations:** Group of Genetics, Breeding and Biochemistry of Brassicas, Misión Biológica de Galicia, Spanish Council for Scientific Research (MBG-CSIC), 36080 Pontevedra, Spain; pvelasco@mbg.csic.es (P.V.); jcfernandez@mbg.csic.es (J.C.F.); ecartea@mbg.csic.es (M.E.C.)

**Keywords:** *Brassica oleracea*, *Brassica rapa*, *Brassica napus*, glucosinolates, phenolic compounds, antioxidant capacity, food quality

## Abstract

*Brassica rapa* is grown in northwestern Spain to obtain turnip greens. The tops of the same plants (flower stems with buds) are cut and sell as turnip tops, increasing the value of the crop. This practice could be extended to other brassicas. The objectives of this work are to study the phytochemical potential of tops of coles (*Brassica oleracea*) and leaf rape (*Brassica napus*) compared to turnip tops and to compare tops of different coles (cabbage, kale, tronchuda cabbage), which differ in their morphology and use. We evaluated the content of glucosinolates and phenolic compounds and the antioxidant capacity in leaves and tops of the three species. We found that tops had higher amount of glucosinolates than leaves. Phenolic content and antioxidant capacity followed the opposite trend. Therefore, consumption of leaves and tops are complementary, since both type of organs are enriched with different types of compound. Local varieties of kale, curly kale, cabbage and curly leave cabbage are interesting because of their GSLs and phenolic content and antioxidant capacity in both leaves and tops. From the human health perspective, tops of coles and leaf rape are interesting as new crops to include in the diet.

## 1. Introduction

The intake of *Brassica* vegetables has benefits on human health. They are good sources of a variety of nutrients and phytochemicals that may work synergistically to help to prevent certain types of chronic diseases, cancer, cardiovascular diseases and immune dysfunction [1]. Glucosinolates (GSLs) are a class of phytochemicals present in the Brassicaceae family. After their intake, they are hydrolyzed in various active products, mainly isothiocyanates (ITCs) which have protective effects in certain forms of cancer (i.e., prostate, intestinal, liver, lung, breast and bladder), chronic inflammation and neurodegeneration [1,2]. Health properties of *Brassica* crops are also related to their antioxidant capacity. In fact, broccoli and kale have more antioxidant capacity than some of the most popular vegetables, such as spinach, potato, carrot, purple onion, green pepper, beet rhubarb and green bean [3,4]. The antioxidant capacity of *Brassica* foods is mainly related to the presence of phenolic compounds [5].

A collection of vegetable *Brassica* crops is currently kept in the Germplasm Bank placed at Misión Biológica de Galicia (MBG-CSIC, Pontevedra, Spain). This collection is composed of local varieties of the three main species cultivated in northwestern Spain. There are 332 registered *Brassica oleracea* varieties, including kale, cabbage and tronchuda cabbage, known generically as coles. Kale and cabbages in the collection show variability in leaf morphology, as some varieties have flat leaves and others curly ones. There is also variability in the size of the crop. In some locations a small type of kale named coias is grown. The local variety called Poio cabbage produces small and axilar heads instead of an apical. Tronchuda cabbages are grown in the south of the region due to the influence of Portugal, where this type of crop is broadly cultivated. The collection of *Brassica rapa* comprises 238 varieties of turnip, turnip greens and turnip tops. There are 63 local varieties of leaf rape (named locally as nabicol), a crop of *Brassica napus* which is cultivated for its leaves. As a reflection of selection made by growers for years, adaptation of varieties to local environmental conditions and inter-varietal crosses, the collection of the three species show a broad amount of morphological [6,7,8], molecular [9,10] and biochemical diversity [11,12,13,14,15].

**Figure 1:**
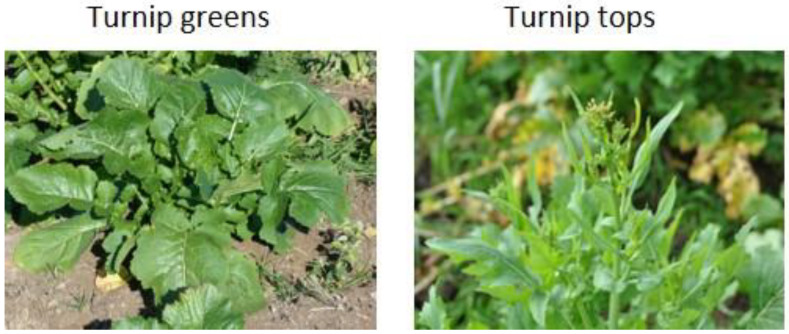
Pictures of turnip greens and tops of *Brassica rapa* cultivated in northwestern Spain.

Varieties are grown according to local practices, mainly in extensive agriculture conditions. Due to their adaptation, few supplies are required to grow them. Adaptation includes tolerance to temperature stress [16] and partial resistance to local pests and diseases [17,18]. Some of these varieties are cultivated to generate a double profit. In the case of kales, the oldest leaves of the plant are employed to supplement the diet of farm animals, such as chickens, rabbits, cows and pigs. The youngest leaves are used for human consumption. In the case of the species *B. rapa*, the same local variety is grown to obtain leaves (turnip greens) and in the flowering period the tops of the plant (flower stems with buds) are cut and sell as another crop (turnip tops) (Figure 1).

In this way, the value of the crop increases and less harvest by-products are produced. The cultivation of turnip greens and turnip tops is well stablished in northwestern Spain, in Portugal and Italy. Both products are highly demanded in the market because of their bitter taste and their highly nutritive properties and sensory quality [19]. Some growers perform the same practice with coles and leaf rape varieties, but the use of tops as a secondary crop is not so extensive as in the case of *B. rapa*. Bioactive compounds and antioxidant capacity of turnip tops have been studied, but these parameters are unknown in cole and leaf rape tops. In order to investigate the healthy properties of tops, it would be interesting to know the concentration of these compounds. The potential of these products as a secondary crop would be increased if the content of phytochemicals was comparable to that of leaves and this information was made available to consumers. These secondary crops could offer a new alternative in the food market, which is always looking for new products. Information on their phytochemical content can satisfy the demand of consumers concerned about these properties.

The objectives of this work are to study the phytochemical potential of cole and leaf rape tops compared to turnip tops, to use these as secondary crops and to compare tops of different local varieties of cole, which differ in their morphology and use.

## 2. Materials and Methods

### 2.1. Plant Material

Twenty varieties corresponding to three *Brassica* species were evaluated in this study (Table 1). Eight local varieties belonged to *B. oleracea* (including different types of kale, cabbage and tronchuda cabbage crops), five local varieties of *B. napus* var. *pabularia* (leaf rape or nabicol) and five varieties of *B. rapa* var. *rapa* (turnip greens and tops) were included. Local varieties are kept at the Gene Bank at MBG-CSIC. Varieties were chosen based on previous studies made at MBG on their morphological and agronomical performance [6,7,8].

### 2.2. Field Trials

Varieties were evaluated during 2019–2020 at Pontevedra, Spain (42°24’ N, 8°38’ W, 20 m.a. s.l) in a representative location of *Brassica* production. Varieties were planted in multi-pot trays, and seedlings were transplanted into the field at the five-six leaf stage. Transplanting dates were 28 June for *B. oleracea* crops and 15 October for *B. napus* and *B. rapa* crops. Transplantation was performed following a randomized complete block design with three replications. Each plot consisted of two rows with ten plants per row. Space between rows was 0.8 m and space between plants was 0.5 m. Plants were watered after transplanting and when required by drip irrigation. Cultural operations, fertilization, and weed control were made according to local practices. Force^®^ (Syngenta, Basel, Switzerland) was added at the time of transplantation to combat soil insects and Pyganic 1,4 (Biograd, Grassobbio (BG)) for aphid control.

Leaf samples were taken in the optimal consumption period of each crop, approximately seven months after transplanting. Top samples were collected from crops on different dates, depending on the formation of buds (Table 1). Five samples of healthy leaves and tops from five plants per plot were used. Samples were frozen on dry ice and kept at −80 °C. Samples were lyophilized (BETA 2–8 LD plus, Christ, GmbH, Osterode am Harz, Germany) for 72 h and then grounded to obtain a fine powderIKA-A10 (IKA-Werke GmbH & Co. KG, Staufen, Germany).

### 2.3. Evaluation of Antioxidant Activity: ABTS Assay

Ten mg of the fine powder were extracted with 1 mL of 80% aqueous methanol in dark maceration for 24 h. After centrifugation of methanolic extracts (3700 rpm, 5 min), the supernatants were employed to determine the antioxidant capacity and phenolic content. To evaluate antioxidant capacity, we employed the ABTS (Sigma–Aldrich Chemie GmbH (Steinheim, Germany) assay following [20] with some modifications. The antioxidant capacity was normalized to Trolox (Sigma–Aldrich Chemie GmbH (Steinheim, Germany) equivalents per gram (g) of dry weight (dw). Absorbances were measured at 734 nm after 30 min of incubation in the dark at room temperature in a microplate spectrophotometer (Spectra MR; Dynex Technologies, Chantilly, VA, USA). Three replications were done for each measurement.

### 2.4. Estimation of Phenolic Content

Methanolic extracts were oxidized with 50 mL of 0.5 M Folin-Ciocalteau reagent (Sigma–Aldrich Chemie GmbH (Steinheim, Germany). After 5 min, 200 mL of a 20% Na_2_CO_3_ solution were added to neutralize the reaction. Then, the absorbance was read at 760 nm in a microplate spectrophotometer (Spectra MR; Dynex Technologies, Chantilly, VA, USA) after 2 h of incubation in the dark at room temperature, following the method described by [21]. Results were expressed as micromoles of gallic acid (Sigma–Aldrich Chemie GmbH (Steinheim, Germany) equivalents per gram of dry weight. Three replications were done for each measurement.

### 2.5. GSLs Identification and Quantification

Sample extraction and de-sulfation were performed following [22]. Chromatographic analyses were performed on an Ultra-High-Performance Liquid-Chromatograph, UHPLC Nexera LC-30AD (Shimadzu, Kyoto, Japan) equipped with a Nexera SIL-30AC injector (Shimadzu, Kyoto, Japan) and one SPD-M20A UV/VIS photodiode array detector (Shimadzu, Kyoto, Japan). The UHPLC column was a XSelect HSS T3 XP Column C18 protected with a C18 guard cartridge (Waters Corporation, Milford, MA, USA). The oven temperature was set at 30 °C. Compounds were separated in aqueous acetonitrile, with a flow of 0.5 mL min^−1^: 1.5 min at 100% H_2_O, an 11 min gradient from 0% to 25% (*v*/*v*) acetonitrile, 1.5 min at 25% (*v*/*v*) acetonitrile, a minute gradient from 25% to 0% (*v*/*v*) acetonitrile, and a final 3 min at 100% H_2_O. Data were recorded with the LabSolutions software (Shimadzu, Kyoto, Japan). All GSLs were quantified at 229 nm. Glucotropaeolin (GTP, monohydrate from Phytoplan, Diehm & Neuberger GmbH, Heidelberg, Germany) was employed as an internal standard to check the quality of GSL extraction. Specific GSLs were identified by comparing retention times and UV spectra with standards (Phytoplan, Diehm & Neuberger GmbH, Heidelberg, Germany). Calibration equations were made with at least five data points for the GSLs glucoiberin (y = 99,397x; *R*^2^ = 0.950), sinigrin (y = 484,871x; *R*^2^ = 0.994), gluconapin (y = 352,910x; *R*^2^ = 0.999), glucobrassicanapin (y = 357,893x; *R*^2^ = 0.997), glucoerucin (y = 276.122x; *R*^2^ = 0.999), glucobrassicin (y = 869,483x; *R*^2^ = 0.988), gluconasturtiin (y = 342,954x; *R*^2^ = 0.997) and progoitrin (y = 398,645x; *R*^2^ = 0.980). GLSs are reported as µmol g^−1^ dry weight (dw). Three replications were done for each measurement.

### 2.6. Statistical Analysis

Analysis of variance was performed for antioxidant capacity, total polyphenol content and GSL content with PROC GLM of SAS v. 9.2. Species, crops, varieties and sample types were considered as fixed effects, whereas replications were considered as random effects. Means comparisons were carried out using Fisher’s protected least significant difference (LSD) at the 0.05 level of probability [23].

## 3. Results

### 3.1. Comparison of GSL Content, Phenolic Content and Antioxidant Capacity between Organs and Among Species

**Figure 2:**
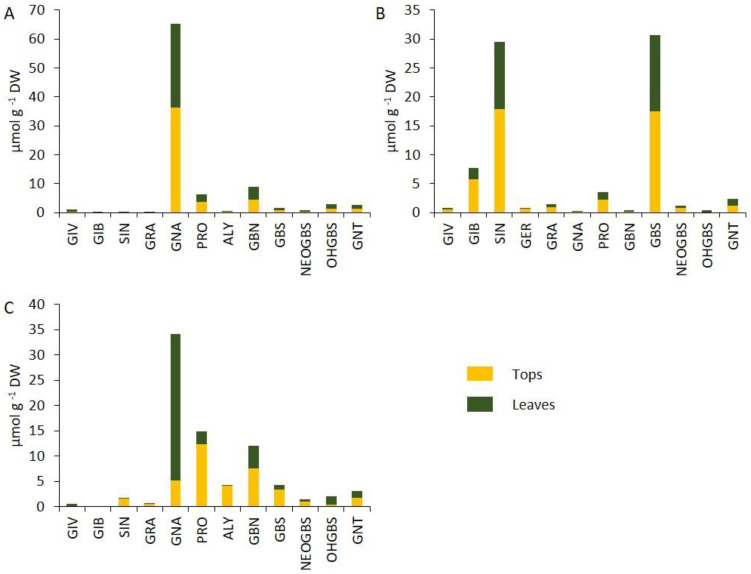
Concentration of individual glucosinolates (GSLs) in tops and leaves of the species (**A**) *B. rapa*, (**B**) *B. oleracea* and (**C**) *B. napus*. Thirteen GSLs were found in the experiment, the aliphatic glucoiberverin (GIV), glucoiberin (GIB), sinigrin (SIN), glucoerucin (GER), glucoraphanin (GRA), gluconapin (GNA), progoitrin (PRO), glucoalyssin (ALY) and glucobrassicanapin (GBN); the indolic glucobrassicin (GBS), neo-glucobrassicin (NEOGBS) and hydroxy-glucobrassicin (OHGBS) and the aromatic gluconasturtiin (GNT).

Thirteen GSLs were detected in the three species. Among aliphatic GSLs we detected the 3C-GSLs glucoiberverin (GIV), glucoiberin (GIB), and sinigrin (SIN); the 4C-GSLs glucoerucin (GER), glucoraphanin (GRA), gluconapin (GNA) and progoitrin (PRO) and the 5C-GSLs glucoalyssin (ALY) and glucobrassicanapin (GBN). ALY was present in *B. rapa* and *B. napus* but not in *B. oleracea*, whereas GER was only found in *B. oleracea* but not in the other species (Figure 2A–C, Supplementary Figure S1). Regarding indolic GSLs, glucobrassicin (GBS), neo-glucobrassicin (NEOGBS) and hydroxy-glucobrassicin (OHGBS) were detected in the three species. The aromatic gluconasturtin (GNT) was present in the three species (Figure 2A–C). Regarding GSLs profile, *B. rapa* was dominated by the presence of GNA in both tops and leaves. The major GSLs of both organs were GBS, SIN and GIB in *B. oleracea* (Figure 2A,B). In the case of *B. napus*, the GSL profile of leaves is dominated by the presence of GNA followed by GBN and PRO, whereas the major GSLs in the tops was PRO, followed by GBN and GNA (Figure 2C). Individual GSLs were found in higher concentration in tops compared to leaves (Figure 2A–C).

**Figure 3:**
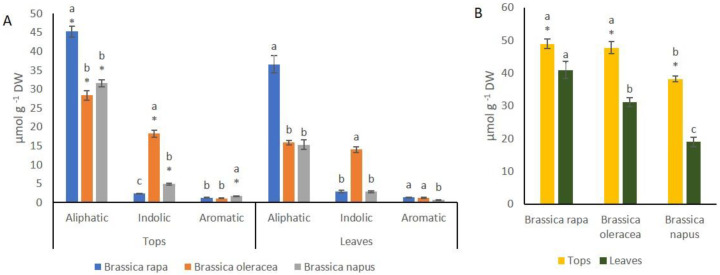
Concentration of glucosinolates (GSLs) of tops and leaves of *B. rapa*, *B. oleracea* and *B. napus*. (**A**) Aliphatic, indolic and aromatic GSL content. (**B**) total GSL content. Letters on the top of bars indicate least significant differences among species at *p* ≤ 0.05. * indicate significant Student’s t between organs of the same species at *p* ≤ 0.05.

Analysis of variance showed that there were significant differences of GSL content between organs (leaves and tops) for each species. Generally speaking, concentration of aliphatic, indolic, aromatic and total amount of GSLs was higher in tops compared to leaves in the three species (Figure 3A,B). Analysis of variance showed significant differences among species for GSL content in tops and leaves. *B. rapa* showed the highest content of aliphatic GSL in both organs compared to the other species (Figure 3A). *B. oleracea* showed the highest content of indolic GSL in both organs, whereas aromatic GSL concentration was significantly higher in tops of *B. napus* compared to the other two species (Figure 3A). *B. rapa* and *B. oleracea* showed similar total GSL content in tops (48.90 and 47.75 µmol g^−1^ DW, respectively) compared to *B. napus* (38.19 µmol g^−1^ DW). *B. rapa* had higher concentration of total GSLs (40.92 µmol g^−1^ DW) in leaves compared to *B. oleracea* (31.78 µmol g^−1^ DW) and *B. napus* (19.95 µmol g^−1^ DW) (Figure 3B).

**Figure 4:**
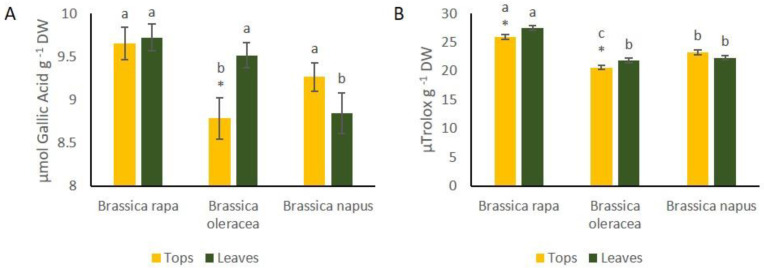
(**A**) Total phenolic content, measured with the Folin-Ciocalteu assay, of tops and leaves of *B. rapa*, *B. oleracea* and *B. napus*. (**B**) Antioxidant capacity, measured with ABTS assays of tops and leaves of *B. rapa*, *B. oleracea* and *B. napus*. Letters on the top of bars indicate least significant differences among species at *p* ≤ 0.05. * indicate significant Student´s t between organs of the same species at *p* ≤ 0.05.

Phenolic content was significantly higher in leaves of *B. oleracea* than in tops, whereas in the other two species there were no differences between organs (Figure 4A). Phenolic content was significantly higher in tops of *B. rapa* compared to the other species (Figure 4A), while phenolic content of leaves did not differ from that of *B. oleracea*. Antioxidant capacity, measured by ABTS assay, was significantly higher in leaves of *B. rapa* and *B. oleracea* than in tops. Leaves and tops of *B. rapa* showed higher ABTS values than those of *B. oleracea* and *B. napus* (Figure 4B).

### 3.2. Comparison of GSLs Content, Phenolic Content and Antioxidant Capacity among Type Crops of B. oleracea

**Figure 5:**
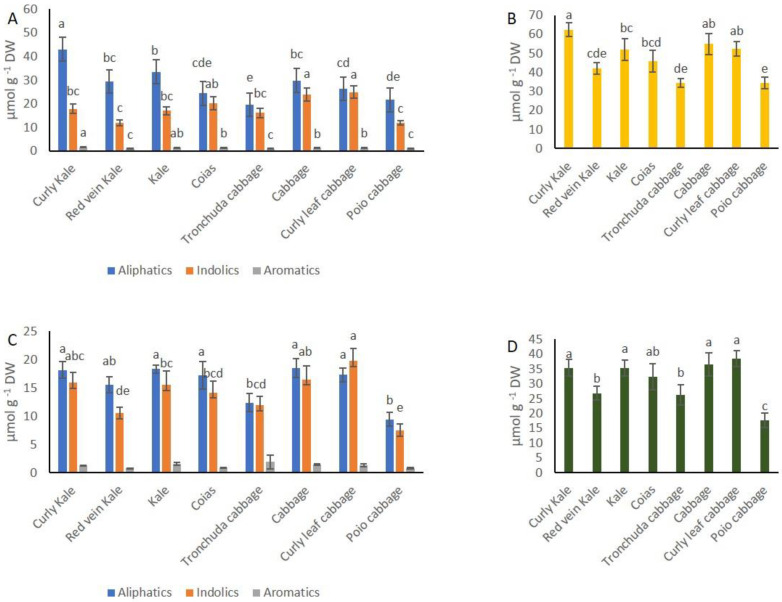
Concentration of glucosinolates (GSLs) of tops and leaves of *B. oleracea* crops. (**A**) Aliphatic, indolic and aromatic GSL content of tops. (**B**) Total GSL content of tops. (**C**) Aliphatic, indolic and aromatic GSL content of leaves. (**D**) total GSL content of leaves. Letters on the top of bars indicate least significant differences among *B. oleracea* crops at *p* ≤ 0.05.

Regarding GSL content of the cole tops, curly kale showed significantly higher amount of aliphatic GSLs than the other crops (Figure 5A). Cabbage, curly leaf cabbage and coias showed significantly higher amount of indolic GSLs in tops than the other varieties. Curly kale and kale had a higher amount of aromatic GSLs than the rest of *B. oleracea* crops (Figure 5A). GSLs in leaves showed a similar trend than that in tops, but lower number of significant differences were found in this organ. Poio cabbage and tronchuda cabbage differed significantly from the rest of *B. oleracea* crops for their low aliphatic GSL content in leaves (Figure 5C). Cabbage, curly leaf cabbage and curly kale had significantly more indolic GSLs than the other crops. There were no differences in content of aromatic GSLs in leaves. Curly kale, cabbage and curly leaf cabbage showed significant higher amount of GSLs than the other *B. oleracea* crops in tops and leaves (Figure 5B,D).

**Figure 6:**
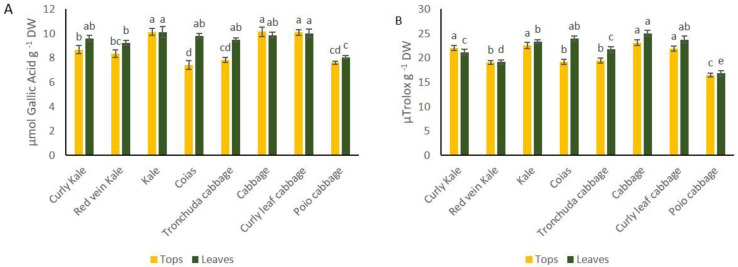
(**A**) Total phenolic content, measured with the Folin-Ciocalteu assay, of tops and leaves of *B. oleracea* crops. (**B**) Antioxidant capacity, measured with ABTS assays of tops and leaves of *B. oleracea* crops. Letters on the top of bars indicate least significant differences among species at *p* ≤ 0.05.

Folin-Ciocalteau assay of *B. oleracea* varieties showed that kale, cabbage and curly leaf cabbage stand out over other crops because they have higher concentration of phenolic compounds in leaves and tops (Figure 6A). The same three crops showed the best antioxidant capacity among all of them (Figure 6B). On the contrary, phenolic content and antioxidant capacity of Poio cabbage was poorer compared to the other crops (Figure 6A, B).

## 4. Discussion

### 4.1. Comparison of GSL Content, Phenolic Content and Antioxidant Capacity between Organs and Among Species

Tops of *B. rapa*, *B. oleracea* and *B. napus* species had a higher amount of GSLs than leaves. Agreeing with other authors, GSL content varies according to the organ under study. Following [24,25], seeds of *B. oleracea* accumulate the highest GSL content, followed by flower buds and vegetative leaves. Differential concentration may reflect the novo synthesis of these compounds and/or the mobilization of the same [26]. Since these compounds have a defensive role in plants, their accumulation first in flower buds and later in seeds may respond to the need to increase the defense system in order to protect these organs against plant pest and pathogens.

Turnip tops are commonly grown and consumed in northwestern Spain mainly in soups and stews. Their agronomical, morphological and quality performance have been studied in depth [15,19,27]. Due to their potential in human health and their bitter taste, they are appreciated in northwestern Spain, where they are commonly used in traditional cuisine. Their cultivation started in the South of Spain after adaptation to dry climatic conditions [28]. The advantage of cultivating turnip tops is that the same crop can be cultivated for two purposes. During the vegetative stage, approximately five months, growers cut the leaves of the crop (turnip greens) and sell them in bundles in supermarkets. Then, in the flowering period, growers cut the tops as a secondary use of the crop. Turnip tops are more expensive, since they can only be obtained in a short period of time (at the beginning of the flowering period). Therefore, growers increase their profit by creating two different uses for the same crop.

Tops of coles and leaf rape could have the same secondary use. Moreover, we have found that tops of the cited species have higher concentration of GSLs than the leaves, which are the organ which is normally consumed. Therefore, from the human health perspective, tops of coles and leaf rape are interesting as new crops to include in the diet.

*B. rapa* and *B. oleracea* did not differ in their total content of GSLs in tops, while *B. rapa* showed the highest content of GSLs in leaves among the three species. Total GSL concentration in tops of the three species is in the range of that detected in broccoli florets by [29], who analyzed 80 broccoli genotypes and found that GSL content ranged between 4.47 and 57.16 µmol g^−1^ DW. Total GSL concentration in leaves of the three species is also in the range of that found in broccoli leaves (0.21 and 1.56 µmol g^−1^ DW) [29]. Regarding the chemical class of GSLs, tops of *B. rapa* have significantly more aliphatic GSLs than tops of the other species, whereas *B. oleracea* tops have more content of indolics and *B. napus* tops have more content of aromatic GSLs than other species. The three species have a different chemical profile of GSLs. Therefore, the consumption of the tops of other species of *Brassica* increases the diversification in the type of GSLs. Profile of each species was coincident with that found in previous research. The profile of *B. rapa* is dominated by the presence of GNA as previously reported [15], while three major GSLs were detected in *B. oleracea*, the aliphatic SIN and GIB and the indolic GBS, agreeing with [13]. The aliphatic GNA, PRO and GBN were predominant in *B. napus,* agreeing with [14]. There were no qualitative differences between GSL profiles of tops and leaves of *B. rapa* and *B. oleracea* and differences were only quantitative. However, GSL profile of tops and leaves of *B. napus* is different.

A high intake of *Brassica* vegetables reduced the risk of age-related illnesses such as cardiovascular disease and of several types of cancer [30,31]. The contribution of *Brassica* vegetables to health improvement is partly associated with their antioxidant capacity. Antioxidant capacity in *Brassica* crops is mostly related to their content of phenolic compounds [12]. We have measured the content of phenolic compounds and antioxidant capacity of tops and leaves of the three studied species. Tops of *B. oleracea* showed significantly lower concentration of phenolic compounds than leaves. Antioxidant capacity was lower in tops of *B. oleracea* and *B. rapa* compared to that of leaves. Phenolic compounds and antioxidant capacity follow the opposite trend than GSL concentration. Both parameters decrease with the change from vegetative to flowering stage. On the contrary [32], found that concentration of phenolic compounds is higher in flower buds than in leaves. Differences may be due to the different material analyzed. Therefore, consumption of leaves and tops of the studied crops are complementary, since both type of organ are enriched with different types of compound.

### 4.2. Comparison of GSL Content, Phenolic Content and Antioxidant Capacity among Type Crops of B. oleracea

Cole crops are regularly consumed in northwestern Spain. For centuries, local farmers have selected characteristics in local varieties, such as shape, colors or size of the consumed organ. In this way, the same crop shows some variations in morphology which may also reflect differences in the content of phytochemicals, interesting for their relation to human health. In this study we have chosen four kale crops, one showing curly leaves, and another with flat leaves. Both types of kale are very popular in northwestern Spain, but the flat type is normally employed for forage. We have also chosen a local kale variety with red veins, suspecting that the color could be associated with a higher antioxidant capacity. The crop called coias is a type of kale, but smaller in size. Two types of cabbage, with curly and flat leaves, were also chosen because both are cultivated broadly in northwestern Spain. Sometimes morphological variation of local varieties is related to a specific location. This is the case of the cabbage called Poio cabbage, which produces small axillar heads instead of a large apical one. Lastly, tronchuda cabbage is cultivated in northwestern Spain due to the influence of the trade with Portugal, where this crop is widely consumed.

Analysis of GSLs showed that the crops curly kale, cabbage and curly leaf cabbage showed a significantly higher amount of GSLs than the other cole crops in both analyzed organs. While curly kale is outstanding because of its amounts of aliphatic and aromatic GSLs compared to the rest of varieties, cabbage and curly leaf cabbage are enriched in indolic GSLs. Therefore, the three crops are interesting from a consumer point of view.

Regarding phenolics content and antioxidant capacity, kale, cabbage and curly leaf cabbage are outstanding compared to the other crops because of the high value for both parameters in leaves and tops. A previous evaluation of antioxidant capacity of *B. oleracea* and *B. napus* crops, [12] found that the antioxidant capacity of kales was the highest among all the crops analyzed, including broccoli, cabbage, cauliflower and nabicol. Antioxidant activity of curly kale was at least 10-fold higher than that of cauliflower and white cabbage in the study carried out by [33]. The antioxidant properties of curly kale are already known. To optimize the consumption of antioxidants from this crop, extracts from the same are included in detox shakes. The other type of kale grown in northwestern Spain, with flat leaves, has the same or more antioxidant capacity in tops and leaves than curly kale. Therefore, this is a very interesting crop to grow and consume due to its antioxidant activity. However, other crops, such as Poio cabbage performed worse due to their lower concentration of GSLs and antioxidant compared to other local varieties.

## 5. Conclusions

In this work we have demonstrated that the tops of coles and leaf rape are suitable to be employed for human consumption due to their high GSLs and phenolic content and antioxidant capacity. In fact, GSL content of tops was higher than that of leaves, which is the organ that is normally consumed in these crops. GSL concentration, phenolic content and antioxidant capacity were in the range of those found in turnip leaves and tops, which we have employed as control in our experiments. By utilizing the tops as a secondary crop, growers can increase their income at the same time as they reduce harvest residues in the fields. Finally, variability found in the local varieties of *B. oleracea* cultivated in northwestern Spain can be employed to obtain tops enriched in GSLs and phenolic compounds. Local varieties of kale, curly kale, cabbage and curly leave cabbage are very interesting because of their GSL content and antioxidant capacity in both leaves and tops.

## Figures and Tables

**Table 1:** Crop, origin and description of varieties studied, types of sample analyzed and number of days from sowing to collecting.

Crop Type	Species	Variety Name	Days to Harvest	
			Leaves	Tops
Curly kale	*B. oleracea*	BRS0027	272	314
Red vein kale	*B. oleracea*	BRS0049	272	300
Kale	*B. oleracea*	BRS0156	272	300
Coias	*B. oleracea*	BRS0468	272	272
Tronchuda cabbage	*B. oleracea*	BRS0226	265	265
Cabbage	*B. oleracea*	BRS0425	272	288
Curly leaf cabbage	*B. oleracea*	BRS0535	272	288
Poio cabbage	*B. oleracea*	BRS0072	265	265
Turnip tops	*B. rapa*	BRS0082	168	221
Turnip tops	*B. rapa*	BRS0184	168	204
Turnip tops	*B. rapa*	BRS0729	168	168
Turnip tops	*B. rapa*	BRS0730	168	204
Turnip tops	*B. rapa*	BRS0731	168	168
Nabicol (leaf rape)	*B. napus*	BRS0063	168	187
Nabicol (leaf rape)	*B. napus*	BRS0085	168	168
Nabicol (leaf rape)	*B. napus*	BRS0337	168	187
Nabicol (leaf rape)	*B. napus*	BRS0037	168	168
Nabicol (leaf rape)	*B. napus*	BRS0110	168	168

## Data Availability

Not applicable.

## Supplementary Materials

The following are available online at https://www.mdpi.com/article/10.3390/foods10122911/s1, Figure S1: UHPLC chromatograms of glucosinolate profiles in tops and leaves of the species *Brassica rapa*, *Brassica oleracea* and *Brassica napus*.
