# Supplementary material for: New Vegetable Brassica Foods: A Promising Source of Bioactive Compounds
Source: Foods. 2021 Nov 24;10(12):2911. doi: 10.3390/foods10122911 (PMC8700788; doi:10.3390/foods10122911)

Figure S1. UHPLC chromatograms of glucosinolate profiles in tops and leaves of the species *Brassica rapa, Brassica oleracea* and *Brassica napus*.

Peaks:

Number	Name	Retention time
1	Glucoiberin	1.298
2	Progoitrin	1.7
3	Glucoraphanin	2.1
4	Sinigrin	2.25
5	Glucoalysin	3.2
6	Gluconapin	3.8
7	Hydroxyglucobrassicin	4.217
8	Glucoiverberin	4.6
9	Glucobrassicin	5
10	Glucotropaeolin (internal standard)	5.166
11	Glucoerucin	5.3
12	Glubrassicin	5.6
13	Gluconasturtin	6.225
14	Neoglucobrassicin	7.213

Brassica rapa

Tops

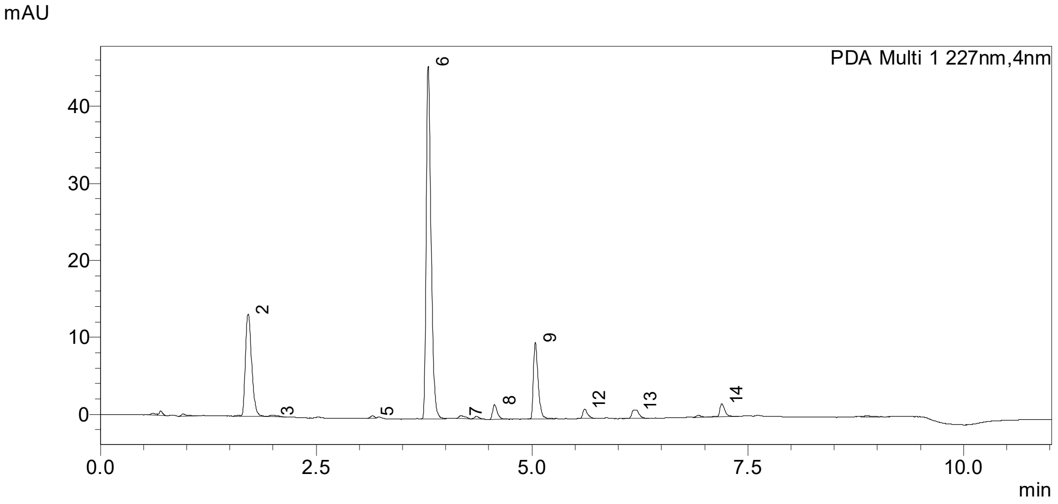

Leaves

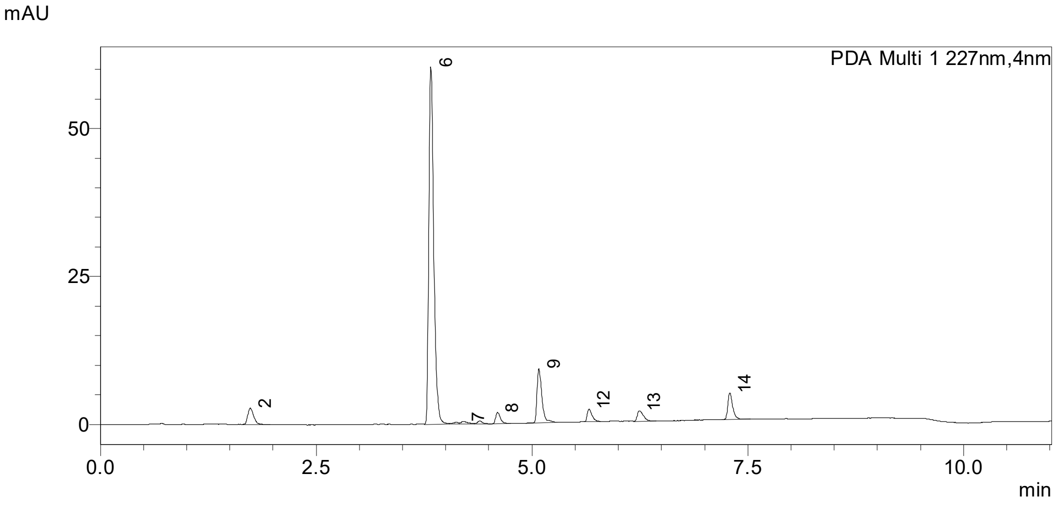

Brassica oleracea

Tops

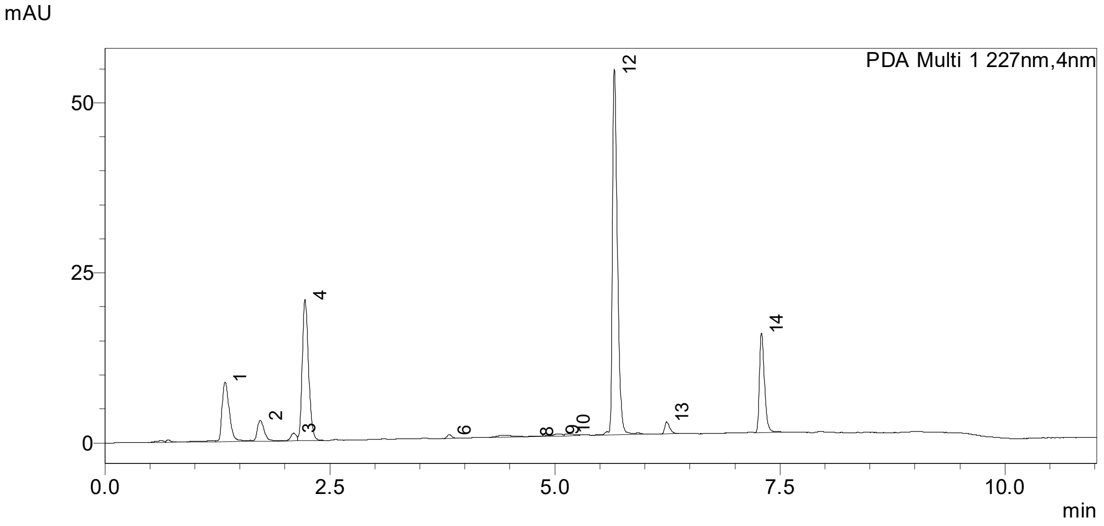

Leaves

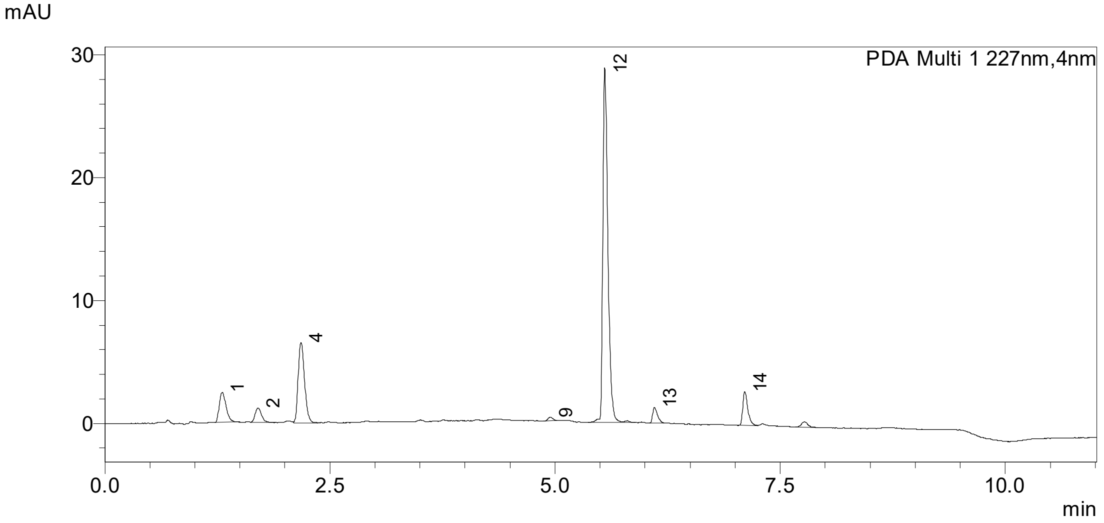

Brassica napus

Tops

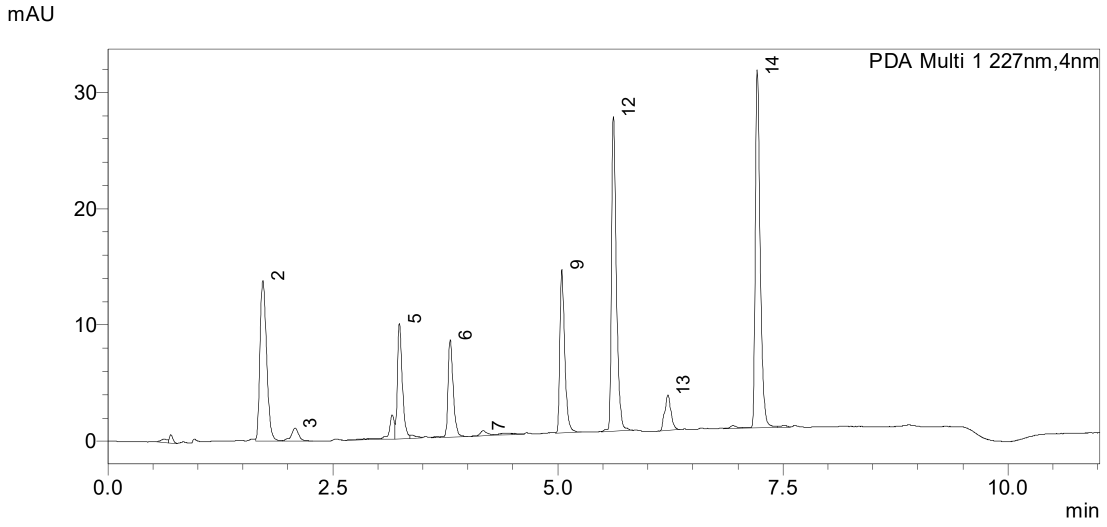

Leaves

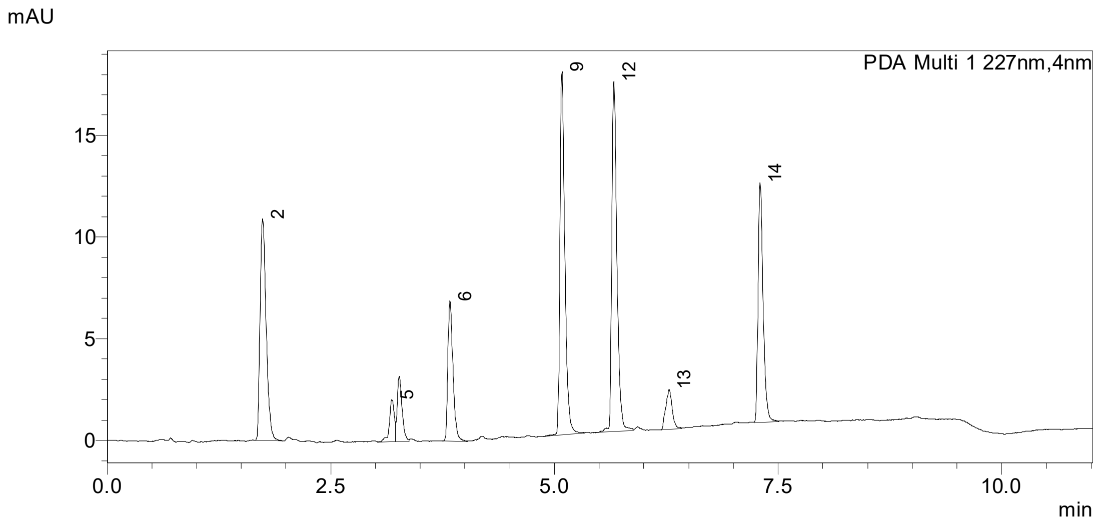

Supplement: Supplementary file 1 [file foods-10-02911-s001.zip › foods-1480551-supplementary.pdf]
